# Integration among national health information systems in Brazil: the case of e-SUS Primary Care

**DOI:** 10.11606/s1518-8787.2021055002931

**Published:** 2021-11-18

**Authors:** Giliate Cardoso Coelho, Rosemarie Andreazza, Arthur Chioro

**Affiliations:** I Universidade Federal de São Paulo Escola Paulista de Medicina São Paulo SP Brasil Universidade Federal de São Paulo. Escola Paulista de Medicina. Programa de Pós-Graduação em Saúde Coletiva. São Paulo, SP, Brasil.

**Keywords:** Electronic Health Records, Control of Forms and Records, eHealth Policies, System Integration Primary Health Care

## Abstract

**OBJECTIVE:**

To measure the degree of integration of the Electronic Citizen’s Record (PEC - *Prontuário Eletrônico do Cidadão*) of the e-SUS Primary Care Strategy (e-SUS AB - *Estratégia e-SUS Atenção Básica*) in the view of other Brazilian´s National Health Information Systems (SNIS - *Sistemas Nacionais de Informação em Saúde*), relating to the internal political-organizational structure of the Brazilian Ministry of Health (MH).

**METHODS:**

This is a qualitative case study. Data collection was carried out through document analysis and semi-structured interviews. In the first stage, we sought to clarify how many SNIS were in use in the Primary Care (PC) of the Unified Health System between 2013 and 2017. Then, we defined as criterion the maintenance of data collection interfaces by the Ministry of Health even after the implementation of the PEC/e-SUS Primary Care in order to measure the integration.

**RESULTS:**

31 SNIS were identified in Primary Care. We observed that 12 of them were completely integrated and 15 presented no unification of interfaces related to PEC/e-SUS Primary Care. Another 4 have partial integration. By correlating these data with the political-organizational structure of the MS, we observed a greater integration with the systems managed by the Department of Primary Care and a persisted fragmentation in SNIS, especially those under the management of the Health Surveillance Department (*Secretaria de Vigilância em Saúde*). The disparity between the integration of the PEC/e-SUS Primary Care with the Health Surveillance SNIS is a sign of the persistence of the division and the false dichotomy between Health Care and Health Surveillance practices and processes in the Ministry of Health – even 30 years after the foundation of the SUS and unification of the state structures of social security hospital care and federal public health in MH.

**CONCLUSION:**

Although still insufficient, the systems integration carried out by the e-SUS Primary Care Strategy, which focuses on reducing user interfaces, can be considered a new fact on the SUS information and information policy agenda.

## INTRODUCTION

In Brazil, health professionals, managers, and researchers live with dozens of health information systems in their work environments, with little or no integration between them. This problem has been reported since the 1980s^1^ and is related to the fragmentation of the State’s bureaucratic structures^2^, the absence of semantic and technological standardization^3–4^, and the low maturity of Information and Communication Technology (ICT) governance policies in organizational services^5^.

Between 2013 and 2018, 54 National Health Information Systems (SNIS - *Sistemas Nacionais de Informação em Saúde*) were identified in operation in Brazil, maintained by the Ministry of Health (MH), including systems for registration, notification of diseases and conditions, control and logistics of supplies and medicines, electronic medical records, laboratory management, accounting control of the production of procedures, among others^6^. Furthermore, they are incorporated into these other systems developed or acquired by states and municipalities.

Often, poor integration requires that the same data be filled in different interfaces, generating rework and increased costs, so turns out to be necessary to maintain several technological solutions with redundant functions. This duplication of records has an impact on the quality of the databases, making analysis and cross-referencing difficult and impacting the reliability of the information produced^4,7^.

Since the creation of the Brazilian Unified Health System (SUS), the state has adopted strategies to improve communication and integration between the SNIS. By the initiative of the Pan American Health Organization, the Inter-Management Network for Health Information (Ripsa - *Rede Intergerencial de Informações para a Saúde*) was created in the late 1990s, intending to produce relevant and integrated information for decision-making in health^8^. In the 2000s, the implementation of the SUS National User Registry (Cadsus - *Cadastro Nacional de Usuário do SUS*) was intended to unify the basic registration information of every Brazilian citizen who uses the SUS. The first information management model of Cadsus provided for the registration, storage, and protection of data as attributions of the municipalities, in a decentralized model that faced serious problems related to duplicate registrations, besides the existence of multiple numbers of the National Health Card (CNS - *Cartão Nacional de Saúde*) for the same user is frequent. This happens when the same user goes to a health service without his card, requiring the reception to make a new registration for him. This problem was only resolved with a structural change in data governance by the centralization of databases in MH. Still, only 14 SNIS were connected to the Cadsus national base in 2014^9^.

In 2013, the MH launched the e-SUS Primary Care Strategy (e-SUS AB - *Estratégia e-SUS Atenção Básica*), One of its objectives has been to promote greater integration between the SNIS operating in the Primary Care Units^10^ by the unification of interfaces data collection in the Simplified Data Collection (CDS - *Coleta de Dados Simplificada*) and in the Electronic Citizens Record (PEC - *Prontuário Eletrônico do Cidadão*) software, developed in partnership with the Universidade Federal de Santa Catarina (UFSC) and made available free of charge to the Municipal Health Departments.

According to the MH, in 2016, data sent to the national e-SUS Primary Care database was carried out by 97% of Brazilian municipalities^11^ and the electronic medical record would already be installed in 9,227 Primary Care Units, corresponding to 21% of the 42.6 thousand existing in 2017^12^. These numbers may vary by region and criteria used. For example, Lima (2018) carried out a diagnostic study on the implementation of the e-SUS Primary Care Strategy in Minas Gerais, concluding that in only 49.1% of the municipalities the strategy had actually been implemented^13^. Moreover, the advanced degree of implementation of the public policy in question seems unequivocal.

When can we say that two or more systems are integrated? In the field of Public Health, this understanding was generally related to the crossing of different bases, increasing data reliability, and producing new health information^14,15^. In turn, the debate on systems integration in the field of health informatics has been around technological solutions aimed at greater automation of communication between systems (mediated by standardized messages and files, syntactic interoperability) or semantic compatibility between the terms used in each of them (semantic interoperability)^16^. However, there is another aspect of systems integration that has been little addressed in academic production and in public health information and informatics policies. We refer to the so-called integration of user interfaces^17^, related to the design and usability of systems by workers and managers in the services responsible for inserting the primary data in the systems.

Focusing on the user-centered, we propose to describe and measure the integration of the PEC with other SUS Primary Care SNIS, relating it to the political-organizational structure of the MS and testing the hypothesis of the systems fragmentation relationship with the bureaucratic structure of the state.

## METHODS

This case study assumes as object the relationship among the e-SUS Primary Care Strategy and the SNIS in Primary Care use, as proposed by Yin (2015)^18^. In the first stage, we sought to clarify how many SNIS were in production in Primary Care between the years 2013 to 2018 – necessary due to the existing conflict between official data sources. The following MH documents related to health information and informatics were analyzed: Information Technology Master Plans (PDTI - *Planos Diretores de Tecnologia da Informação*) from 2014 to 2015^19^, 2016^20^ and 2017 2018^21^; National Policy on Health Information and Informatics (PNIIS - *Política Nacional de Informação e Informática em Saúde*) published in 2016^22^; Datasus Management Report (*Relatório de Gestão do Datasus*) from 2011 a 2014^2323^; Terms of Reference of the processes related to the electronic auction of contract No. 22/2014 (*Termos de Referência dos processos referentes ao pregão eletrônico do contrato*) regarding the contracting of a software factory to provide information technology services for the development and maintenance of information systems^24^; finally, Notice No.01 / 2017 in relation to the computerization program of the Primary Care Units^25^.

The responses of the MS motivated by two requests for access to information based on Law 12,527/2011 (Access to Information Law) were also analyzed, where it was asked about the list of systems in production in MH (nº 25820004082201795) and in Primary Care (nº 25820005771201806). The complete content of the responses can be accessed openly on the page of the Electronic Information System to Citizen (e-SIC - *Sistema Eletrônico de Informações ao Cidadão*)^a^.

In the second stage, the objective was to understand the situation of the integration of each of the SNIS in operation in Primary Care with the Electronic Citizen’s Record (PEC) of the e-SUS Primary Care, clarifying whether the integration actions allowed workers and local managers to discontinue the use of the respective SNIS. Three key informants were interviewed: two members of the development and management team of the e-SUS Primary Care Strategy in the period from 2013 to 2017 and one member of the technical team of a national entity representing the municipal health secretariats. The analysis also included contributions from the experience of one of the researchers in the daily use of PEC/e-SUS Primary Care and other SNIS from his work as a physician in a basic health unit in Rio de Janeiro. Only those where there was consensus among the above sources were considered as integrated SNIS.

The following typology was created to classify the situation of integration between systems:

- Integrated or unified user interfaces (full integration): When two or more systems become invisible to the final user. This means that the user cannot perceive that there is more than one system in operation, because the interfaces are fully integrated.
- Partially integrated or unified user interfaces (incomplete integration): It happens when integration initiatives between the interfaces were identified, but the need for professionals and managers continues using the two systems in their daily lives, given technical, political, or administrative reasons.
- No integration or unification of user interfaces: it happens when no action has been planned or taken for integration between system interfaces.

Finally, we analyzed the degree of integration of the SNIS with the internal organization chart of the MH. In the period from 2013 to 2018, the formal structure of the top management of the agency remained stable, with the existence of seven Secretariats^26^: Health Care (SAS), Executive (SE), Health Surveillance (SVS), Strategic and Participatory Management (SGEP - *Gestão Estratégica e Participativa*), Indigenous Health Special (SESAI - *Especial de Saúde Indígena*), Work Management and Health Education (SGTES - *Gestão do Trabalho e Educação na Saúde*) and Science Technology and Strategic Inputs (SCTIE - *Ciência Tecnologia e Insumos Estratégicos*). The Primary Care Department (DAB - *Departamento de Atenção Básica*), the main sector responsible for formulating and managing the e-SUS Primary Care Strategy, was linked to SAS.

The research was presented and approved by the Ethics Committee of the São Paulo da Universidade Federal de São Paulo Hospital (Unifesp), opinion nº 2.179.195. Respondents signed an Informed Consent Form (FICF) and the interviews were conducted and recorded using the Skype ® software.

## RESULTS

A total of 31 SNIS were found in operation in Primary Care between 2013 and 2018, most of them under the technical management of the SAS or SVS (Box).

**Box:** National Health Information Systems (SNIS) that collected data in Primary Health Care between 2013 and 2018 (except for the e-SUS Primary Care Strategy software).

Abbreviation	System name	Data collected and/or consumed or main purpose in Primary Care	MH Secretariat
BFA	System of the *Bolsa Família* in Health Program	Registration of Anthropometry of *Bolsa Família* Program beneficiaries	SAS
CADSUS	SUS User Registration System	Registration of SUS users	SE
CMD	Minimum Data Set	Registration of outpatient activities and procedures	SAS
CNES	National Register of Health Establishments (*Cadastro Nacional dos Estabelecimentos de Saúde*)	Registration of health establishments	SAS
Fique sabendo	HIV, Syphilis and Viral Hepatitis Rapid Tests control system for strategic actions	Logistic control of quick tests	SVS
GAL	Laboratory Environment Management System (*Sistema de Gestão de Ambiente Laboratorial*)	Visualization of laboratory test results for diseases of public health interest	SVS
Hiperdia	Registration and Monitoring System for Hypertensive and Diabetics	Monitoring of patients with Hypertension and Diabetes	SAS
HORUS	National Pharmaceutical Assistance System	Logistic control of drugs from the basic component of the National Pharmaceutical Assistance Policy	SVS
PMAQ-AB	National Program for Improving Access and Quality of Primary Care. (*Programa Nacional de Melhoria do Acesso e da Qualidade da Atenção Básica*)	Data from programmatic actions and other information from Primary Care for the purpose of evaluating the teams	SAS
Painel-PSE	Adhesion System to the School Health Program	Monitoring of activities carried out by Primary Care teams under the School Health Program	SAS
RAAS-AD	Register of Home Care Health Actions (*Registro das Ações de Saúde da Atenção Domiciliar*)	Registration of home care procedures under the *Melhor em Casa* Program	SAS
RESP	Response to Public Health Events (*Resposta a Eventos de Saúde Pública*)	Notification of microcephaly cases	SVS
SI-PNI	National Immunization Program Information System (*Sistema de informação do Programa Nacional de Imunização*)	Monitoring of vaccine coverage and logistical control of immunobiologicals	SVS
SIA	Outpatient Information System (*Sistema de informações ambulatoriais*)	Record of outpatient procedures	SAS
SIAB	Primary Care Information System (*Sistema de Informação da Atenção Básica*)	Registration of activities and procedures	SAS
SIASI	Indigenous Health Information System	Demography and clinical and administrative monitoring of the indigenous population	SESAI
SICLOM	Clinical and laboratory control and monitoring system for patients undergoing hepatitis treatment	Prescription of antiretroviral drugs for the treatment of HIV/AIDS	SVS
SIM	Mortality Information System (*Sistema de Informação de Agravos e Notificação*)	Natural death record	SVS
SINAN Net	Notifiable Diseases Information System(*Sistema de Informação de Agravos e Notificação*).	Notification of diseases and injuries of public health interest	SVS
SINAN Dengue/ Chikungunya	Information System of Notifiable Diseases for Dengue and Chikungunya (*Sistema de Informação de Agravos de Notificação para Dengue e Chikungunya*)	Notification of cases of dengue and Chikungunya fever	SVS
SINAN Influenza	Disease Information and Notification System – Influenza (*Sistema de Informação de Agravos e Notificação – Influenza*)	Notification of cases of Severe Acute Respiratory Syndrome	SVS
SINASC	Live Birth Information System (*Sistema de Informações sobre Nascidos Vivos*)	Live birth registration	SVS
SIRAM	Microcephaly Child Care Registration System (*Sistema de Registro de Atendimento às Crianças com Microcefalia*)	Recording of care data on children diagnosed with microcephaly	SAS
SISCAN	Cancer Information System (*Sistema de Informação do Câncer*)	Request and results of exam orders related to cancer diagnosis	SAS
SISCEL	CD4 and Viral Load Laboratory Test Control System (*Sistema de Controle de Exames Laboratoriais de CD4 e Carga Viral*)	Orders for CD4 and Viral Load Tests for HIV Patients	SVS
SISREG	National Regulation System (*Sistema Nacional de Regulação*)	Request and/or scheduling of procedures in specialized care	SAS
SIS Pré-natal	Prenatal, Childbirth, Puerperium and Child Monitoring and Evaluation Information System	Monitoring of pregnant women, mothers and newborns	SAS
SISVAN	Nutritional Surveillance and Monitoring System (*Sistema de Vigilância e Acompanhamento Nutricional*)	Monitoring of nutritional status and food consumption	SAS
SIVEP DDA	Computerized System for the Epidemiological Surveillance of Acute Diarrheal Diseases (*Sistema Informatizado de Vigilância Epidemiológica de Doenças Diarreicas Agudas*)	Notification of Acute Diarrheal Disease Cases	SVS
SIVEP Malaria	Malaria case notification Epidemiological Surveillance Information System	Malaria case notification	SVS
Telehealth/Smart	Telehealth Program Results Monitoring and Evaluation System	Control, monitoring and national evaluation of telehealth programs	SGTES

MS: Brazilian Ministry of Health; BFA: System of the *Bolsa Família* in Health Program; CADSUS: User Registration System; CMD: Minimum Data Set; CNES: National Register of Health Establishments; Fique sabendo: HIV, Syphilis and Viral Hepatitis Rapid Tests control system for strategic actions; GAL: Laboratory Environment Management System; Hiperdia: Registration and Monitoring System for Hypertensive and Diabetics; HORUS: National Pharmaceutical Assistance System; PMAQ-AB: National Program for Improving Access and Quality of Primary Care; Painel-PSE: Adhesion System to the School Health Program; RAAS-AD: Register of Home Care Health Actions; RESP: Response to Public Health Events; SI-PNI: National Immunization Program Information System; SIA: Outpatient Information System; SIAB: Primary Care Information System; SIASI: Indigenous Health Information System; SICLOM: Clinical and laboratory control and monitoring system for patients undergoing hepatitis treatment; SIM: Mortality Information System; SINAN Net: Notifiable Diseases Information System; SINAN Dengue/Chikungunya: Information System of Notifiable Diseases for Dengue and Chikungunya; SINAN Influenza: Disease Information and Notification System; SINASC: Live Birth Information System; SIRAM: Microcephaly Child Care Registration System; SISCAN: Cancer Information System; SISCEL: CD4 and Viral Load Laboratory Test Control System; SISREG: National Regulation System; SIS Pré-natal: Prenatal, Childbirth, Puerperium and Child Monitoring and Evaluation Information System; SISVAN: Nutritional Surveillance and Monitoring System; SIVEP DDA: Computerized System for the Epidemiological Surveillance of Acute Diarrheal Diseases; SIVEP Malaria: Malaria case notification Epidemiological Surveillance Information System; Telehealth/Smart: Telehealth Program Results Monitoring and Evaluation System; MH: Brazilian Ministry of Health; SAS: Health Care Secretariat; SE: Executive Secretariat; SVS: Health Surveillance Secretariat; SESAI: Indigenous Health Special Secretariat; SGTES: Work Management and Health Education Secretariat; SCTIE: Science Technology and Strategic Inputs Secretariat.

It was found that the e-SUS Primary Care Strategy performed complete unification of user interfaces with 12 of the 31 nationally-based SNIS in use in Primary Care and incomplete integrations with four SNIS. Another 15 SNIS had no degree of integration, as shown in Table 1.

**Table 1:** Integration situation of e-SUS Primary Care user interfaces with other SNIS.

Integration status	N SNIS	%	List of SNIS
Complete	12	35.50%	CADSUS, CMD, Hiperdia, PMAQ-AB, Painel-PSE, RAAS-AD, SIA, SIAB, SIRAM, SIS Pré-Natal, SISVAN, BFA.
Incomplete	4	16.10%	CNES, HORUS, SI-PNI, SISREG.
None	15	48.40%	Fique Sabendo, GAL, RESP, SIASI, SICLOM, SIM, SINAN Dengue/Chikungunya, SINAN Influenza, SINAN NET, SINASC, SISCAN, SISCEL, SIVEP Malária, SIVEP DDA, Telehealth/SMART.

SNIS: National Health Information Systems; BFA: System of the *Bolsa Família* in Health Program; CADSUS: User Registration System; CMD: Minimum Data Set; CNES: National Register of Health Establishments; Fique sabendo: HIV, Syphilis and Viral Hepatitis Rapid Tests control system for strategic actions; GAL: Laboratory Environment Management System; Hiperdia: Registration and Monitoring System for Hypertensive and Diabetics; HORUS: National Pharmaceutical Assistance System; PMAQ-AB: National Program for Improving Access and Quality of Primary Care; Painel-PSE: Adhesion System to the School Health Program; RAAS-AD: Register of Home Care Health Actions; RESP: Response to Public Health Events; SI-PNI: National Immunization Program Information System; SIA: Outpatient Information System; SIAB: Primary Care Information System; SIASI: Indigenous Health Information System; SICLOM: Clinical and laboratory control and monitoring system for patients undergoing hepatitis treatment; SIM: Mortality Information System; SINAN Net: Notifiable Diseases Information System; SINAN Dengue/Chikungunya: Information System of Notifiable Diseases for Dengue and Chikungunya; SINAN Influenza: Disease Information and Notification System; SINASC: Live Birth Information System; SIRAM: Microcephaly Child Care Registration System; SISCAN: Cancer Information System; SISCEL: CD4 and Viral Load Laboratory Test Control System; SISREG: National Regulation System; SIS Pré-natal: Prenatal, Childbirth, Puerperium and Child Monitoring and Evaluation Information System; SISVAN: Nutritional Surveillance and Monitoring System; SIVEP DDA: Computerized System for the Epidemiological Surveillance of Acute Diarrheal Diseases; SIVEP Malaria: Malaria case notification Epidemiological Surveillance Information System; Telehealth/Smart: Telehealth Program Results Monitoring and Evaluation System.

Integration varied according to the internal sector of the MS responsible for the technical management of the system (Table 2). Although the SAS and SVS have similar numbers of SNIS in use in Primary Care, there was an important disparity between the integration of their respective systems with the e-SUS Primary Care.

**Table 2:** Integration of e-SUS with other SNIS by MS Secretariat. Organization chart for September 2018.

MH Secretariat	Total SNIS in use Primary Care	SNIS integration with e-SUS Primary Care		
		Complete	Incomplete	none
SAS	14	11	2	1
SVS	13	0	1	12
SE	1	1	0	0
SCTIE	1	0	1	0
SESAI	1	0	0	1
SGTES	1	0	0	1
SGEP	0	0	0	0
TOTAL	31	12	4	15

MH: Brazilian Ministry of Health; SNIS: National Health Information Systems; SAS: Health Care Secretariat; SE: Executive Secretariat; SVS: Health Surveillance Secretariat; SGEP: Strategic and Participatory Management Secretariat; SESAI: Indigenous Health Special Secretariat; SGTES: Work Management and Health Education Secretariat; SCTIE: Science Technology and Strategic Inputs Secretariat.

## DISCUSSION

The SIS fragmentation is a phenomenon reported by several authors when analyzing health information policies in different regions of the world^27–31^, but we did not find studies that dimensioned such fragmentation, based on the counting and description of the SNIS in operation. The record of 31 SNIS in use in Primary Care from 2013 to 2018 shows that the trend of fragmentation persists in the country, documented in Brazil in several technical studies^32–34^.

In countries that promoted projects to change this scenario, the focus has been on better integration between national databases^35–37^ or the establishment of semantic and technological standards for recording and exchanging information between different systems^38,39^. No describing studies were about SNIS integration, focusing on the user interfaces.

When we analyze the result of the integration carried out by e-SUS Primary Care and the political-organizational structure of the MH, we noted that there was greater integration with the SNIS under the direct management of the DAB (Figure) and a low integration with systems from other secretariats beyond of SAS itself.

**Figure:**
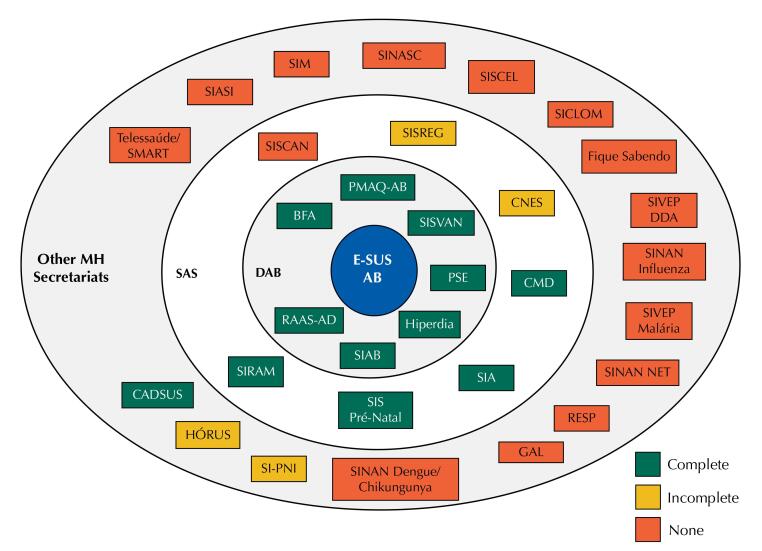
Integration of e-SUS Primary Care interfaces with SNIS used in Primary Care as per the MH internal organization chart.

MS: Brazilian Ministry of Health; SAS: Health Care Secretariat; DAB: Primary Care Department; BFA: System of the *Bolsa Família* in Health Program; CADSUS: User Registration System; CMD: Minimum Data Set; CNES: National Register of Health Establishments; E-SUS AB: e-SUS Primary Care; Fique sabendo: HIV, Syphilis and Viral Hepatitis Rapid Tests control system for strategic actions; GAL: Laboratory Environment Management System; Hiperdia: Registration and Monitoring System for Hypertensive and Diabetics; HORUS: National Pharmaceutical Assistance System; PMAQ-AB: National Program for Improving Access and Quality of Primary Care; Painel-PSE: Adhesion System to the School Health Program; RAAS-AD: Register of Home Care Health Actions; RESP: Response to Public Health Events; SI-PNI: National Immunization Program Information System; SIA: Outpatient Information System; SIAB: Primary Care Information System; SIASI: Indigenous Health Information System; SICLOM: Clinical and laboratory control and monitoring system for patients undergoing hepatitis treatment; SIM: Mortality Information System; SINAN Net: Notifiable Diseases Information System; SINAN Dengue/Chikungunya: Information System of Notifiable Diseases for Dengue and Chikungunya; SINAN Influenza: Disease Information and Notification System; SINASC: Live Birth Information System; SIRAM: Microcephaly Child Care Registration System; SISCAN: Cancer Information System; SISCEL: CD4 and Viral Load Laboratory Test Control System; SISREG: National Regulation System; SIS Pré-natal: Prenatal, Childbirth, Puerperium and Child Monitoring and Evaluation Information System; SISVAN: Nutritional Surveillance and Monitoring System; SIVEP DDA: Computerized System for the Epidemiological Surveillance of Acute Diarrheal Diseases; SIVEP Malaria: Malaria case notification Epidemiological Surveillance Information System; Telehealth/Smart: Telehealth Program Results Monitoring and Evaluation System

In the case of integration among SVS SNIS, including the Epidemiological Surveillance, Care for the Population with HIV/AIDS and Hepatitis, birth, and mortality monitoring and immunization policies, we observed only a partial advance in the integration of the e-SUS Primary Care with the SI-PNI.

The most worrisome case seemed to be the SNIS of the Department of HIV/AIDS, not even mentioned in the list of SNIS in operation in Primary Care in the MH bidding for Primary Care Units computerization^25^.

The disparity between the integration of e-SUS Primary Care with the SVS and SAS SNIS can be considered a sign that some degree of historical division and false dichotomy between Health Care and Health Surveillance practices and processes still persists^40^, even after 30 years since the foundation of the SUS and unification of the state structures of social security hospital care and federal public health in MH.

The governability of the managing area of e-SUS Primary Care over other SNIS proved to be a variable to be considered in the integration process, as there was a higher success rate of integration of e-SUS Primary Care with DAB’s own SNIS when compared to the SNIS under the management of other MH departments and secretariats.

The frequency and intensity of the use of SNIS in health services was a lesser factor, given that SNIS with intense use at the local level of the SUS had incomplete or not started integration, such as the Sisreg, Horus, and Sinan Net.

## CONCLUSION

Considering the complexity of integration between legacy systems in organizations, it is reasonable that after five years of the e-SUS Primary Care Strategy implementation, the operation of SNIS in Primary Care is just partially integrated. Although still insufficient, the integration of systems carried out with about ⅓ of the SNIS in operation in Primary Care, focused on reducing user interfaces, is a new fact on the agenda of the SUS information and information policy.

The relative uniqueness of the Brazilian experience of integration between health information systems is remarkable. Brazil promoted the creation of a state electronic medical record as a kind of “hub” for other SNIS created by the government, more specifically by the federal sphere. Although we believe that the “developmentalist” stance taken by the Brazilian state contributes to the expansion of computerization of primary care in the country, we pay attention to the need for greater integration of the large volume of market software in use by states and municipalities in the national SNIS bases, following the trend of investment in public regulation policies associated with greater semantic and technological interoperability between systems, as observed in other countries.

## Footnotes

a Controladoria-Geral da União (BR). Sistema Eletrônico do Serviço de Informação (e-SIC) e Fala.BR – Módulo de Acesso à Informação: acesso à informação e ouvidoria em uma única plataforma. Brasília, DF; 2021 [cited 2021 oct 20]. Available from: http://esic.cgu.gov.br
