# Supplementary material for: Integration among national health information systems in Brazil: the case of e-SUS Primary Care
Source: Rev Saude Publica. 2021 Nov 18;55:93. doi: 10.11606/s1518-8787.2021055002931 (PMC8659614; doi:10.11606/s1518-8787.2021055002931)
Supplement: Translation

# Translation: Integração entre os sistemas nacionais de informação em saúde: o caso do e-SUS Atenção Básica

**Authors:** Giliate Cardoso Coelho, Rosemarie Andreazza, Arthur Chioro

**Affiliations:** I São Paulo SP Brasil Universidade Federal de São Paulo. Escola Paulista de Medicina. Programa de Pós-Graduação em Saúde Coletiva. São Paulo, SP, Brasil.

**Keywords:** Registros Eletrônicos de Saúde, Controle de Formulários e Registros, Políticas de eSaúde, Atenção Primária à Saúde, Integração de Sistemas

## Abstract

**OBJETIVO:**

Medir o grau de integração do Prontuário Eletrônico do Cidadão (PEC) da Estratégia e-SUS Atenção Básica (e-SUS AB) com outros Sistemas Nacionais de Informação em Saúde (SNIS), o relacionando à estrutura político-organizacional interna do Ministério da Saúde (MS).

**MÉTODOS:**

Trata-se de um estudo de caso de caráter qualitativo. A coleta de dados foi realizada através de análise documental e entrevistas semiestruturadas. Na primeira etapa buscou-se esclarecer quantos SNIS estiveram em uso na Atenção Básica do Sistema Único de Saúde entre 2013 e 2017. Em seguida, para medir a integração, foi aplicado como critério a manutenção das interfaces de captação de dados pelo Ministério da Saúde, mesmo após a implantação do PEC/e-SUS AB.

**RESULTADOS:**

Foram identificados 31 SNIS na Atenção Básica. Observou-se que 12 deles foram completamente integrados e em 15 não houve nenhuma unificação de interfaces com o PEC/e-SUS AB. Outros 4 tiverem integração parcial. Ao correlacionar esses dados com a estrutura político-organizacional do MS, verificou-se uma maior integração com os sistemas geridos pelo Departamento de Atenção Básica e uma persistência da fragmentação com os SNIS, especialmente aqueles sob gestão da Secretaria de Vigilância em Saúde. A disparidade entre a integração do PEC/e-SUS AB com os SNIS da Vigilância em Saúde é um sinal da persistência da divisão e da falsa dicotomia entre práticas e processos de Assistência à Saúde e Vigilância em Saúde no Ministério da Saúde – mesmo após 30 anos da fundação do SUS e unificação das estruturas estatais da assistência hospitalar previdenciária e da saúde pública federal no MS.

**CONCLUSÃO:**

Apesar de ainda insuficiente, a integração de sistemas efetivada pela Estratégia e-SUS AB, que tem foco na redução de interfaces de usuário, pode ser considerada um fato novo na agenda da política de informação e informática do SUS.

## INTRODUÇÃO

Os profissionais, gestores e pesquisadores da área da saúde convivem com dezenas de sistemas de informação em saúde em seus ambientes de trabalho, com pouca ou nenhuma integração entre si. É um problema relatado desde a década de 1980^1^ e está relacionado à fragmentação das estruturas burocráticas do Estado^2^, à ausência de padronização semântica e tecnológica^3–4^ e à baixa maturidade das políticas de governança de Tecnologia de Informação e Comunicação (TIC) nas organizações^5^.

Entre 2013 e 2018, foram identificados 54 Sistemas Nacionais de Informação em Saúde (SNIS) em funcionamento no Brasil, mantidos pelo Ministério da Saúde (MS), incluindo sistemas de cadastro, notificação de doenças e agravos, controle e logística de insumos e medicamentos, prontuários eletrônicos, gestão laboratorial, controle contábil da produção de procedimentos, dentre outros^6^. Ademais, incorporam-se a esses outros sistemas desenvolvidos ou adquiridos por estados e municípios.

Frequentemente, a pouca integração exige que os mesmos dados sejam preenchidos em diferentes interfaces, gerando retrabalho e aumento de custos, pois se torna necessário manter várias soluções tecnológicas com funções redundantes. Essa duplicidade de registros tem impacto na própria qualidade das bases de dados, dificultando as análises, os cruzamentos e impactando na confiabilidade da informação produzida^4,7^.

Desde a criação do Sistema Único de Saúde (SUS), algumas estratégias vêm sendo adotadas para melhorar a comunicação e integração entre os SNIS. Por iniciativa da Organização Pan-americana de Saúde, foi criada no final da década de 1990 a Rede Intergerencial de Informações para a Saúde (Ripsa), que tinha como objetivo produzir informações relevantes e integradas para a tomada de decisão na saúde^8^. A implantação do Cadastro Nacional de Usuário do SUS (Cadsus) nos anos 2000 se propunha a unificar as informações cadastrais básicas de todo cidadão brasileiro usuário do SUS. O primeiro modelo de gestão das informações do Cadsus previu o cadastramento, armazenamento e proteção dos dados como atribuições dos municípios, num modelo descentralizado que enfrentou graves problemas relacionados a cadastros duplicados, não sendo incomum a existência de múltiplos números do Cartão Nacional de Saúde (CNS) para o mesmo usuário. Isso acontecia quando o usuário comparecia a um serviço de saúde sem o seu cartão, exigindo que a recepção fizesse um novo cadastro para ele. Esse problema somente foi resolvido quando houve uma mudança estrutural na governança dos dados por meio da centralização das bases no MS. Ainda assim, em 2014, existiam apenas 14 SNIS conectados à base nacional do Cadsus^9^.

Em 2013, o MS lançou a Estratégia e-SUS Atenção Básica (e-SUS AB), que tinha como um de seus objetivos promover maior integração entre os SNIS em funcionamento nas Unidades Básicas de Saúde (UBS)^10^ por meio da unificação das interfaces de captação de dados nos softwares Coleta de Dados Simplificada (CDS) e no Prontuário Eletrônico do Cidadão (PEC), desenvolvidos em parceria com a Universidade Federal de Santa Catarina (UFSC) e disponibilizados gratuitamente às Secretarias Municipais de Saúde.

Segundo o MS, em 2016, o envio de dados para a base nacional do e-SUS AB era realizado por 97% dos municípios brasileiros^11^ e o prontuário eletrônico já estaria instalado em 9.227 UBS, correspondendo a 21% das 42,6 mil existentes em 2017^12^. Esses números podem variar de acordo com a região e critérios utilizados. Por exemplo, Lima (2018) realizou estudo diagnóstico sobre a implantação da Estratégia e-SUS AB em Minas Gerais, concluindo que em apenas 49,1% dos municípios a estratégia havia sido de fato implantada^13^. A par da controvérsia, parece inequívoco o avançado grau de implantação da política pública em questão.

Quando podemos afirmar que dois ou mais sistemas encontram-se integrados? No campo da Saúde Coletiva, esse entendimento geralmente esteve relacionado ao cruzamento de diferentes bases, aumentando a confiabilidade dos dados e produzindo novas informações em saúde^14,15^. Por sua vez, o debate sobre integração de sistemas no campo da informática em saúde tem acontecido em torno das soluções tecnológicas visando maior automação da comunicação entre sistemas (mediada por mensagens e arquivos padronizados, a interoperabilidade sintática) ou da compatibilização semântica entre os termos utilizados em cada um deles (a interoperabilidade semântica)^16^. Todavia, existe um outro aspecto da integração de sistemas que tem sido pouco abordado nas produções acadêmicas e nas políticas públicas de informação e informática em saúde, a chamada integração de interfaces de usuário^17^, relacionada ao design e usabilidade dos sistemas por trabalhadores e gestores nos serviços de saúde, responsáveis pela inserção dos dados primários nos sistemas.

Será a partir desse foco no utilizador-centrado que este artigo se propõe a descrever e medir a integração do PEC com outros SNIS da Atenção básica do SUS, o relacionando à estrutura político-organizacional do MS, testando a hipótese da relação da fragmentação de sistemas com a estrutura burocrática do Estado.

## MÉTODOS

Realizou-se um estudo de caso que toma como objeto a relação entre a Estratégia e-SUS AB e os SNIS em uso na Atenção Básica, nos moldes propostos por Yin (2015)^18^. Na primeira etapa, buscou-se esclarecer quantos SNIS estiveram em produção na Atenção Básica entre os anos 2013 a 2018 – necessário devido ao conflito existente entre as fontes de dados oficiais). Foram analisados os seguintes documentos do MS relacionados à informação e informática em saúde: Planos Diretores de Tecnologia da Informação (PDTI) dos anos de 2014 a 2015^19^, 2016^20^ e 2017 2018^21^; Política Nacional de Informação e Informática em Saúde (PNIIS) publicada em 2016^22^; Relatório de Gestão do Datasus de 2011 a 2014^23^; Termos de Referência dos processos referentes ao pregão eletrônico do contrato nº 22/2014 referente à contratação de fábrica de software para prestação de serviços de tecnologia da informação para o desenvolvimento e a manutenção de sistemas de informação^24^ e, por fim, o Edital nº 01/2017 referente ao programa de informatização das Unidades Básicas de Saúde^25^.

Também foram analisadas as respostas do MS motivadas por dois pedidos de acesso à informação baseados na Lei nº 12.527/2011, onde foi perguntado sobre a lista de sistemas em produção no MS (Protocolo nº 25820004082201795) e na Atenção Básica (Protocolo nº 25820005771201806). O conteúdo completo das respostas pode ser acessado de forma aberta na página do Sistema Eletrônico de Informações ao Cidadão (e-SIC)^a^.

Na segunda etapa, objetivou-se compreender a situação da integração de cada um dos SNIS em funcionamento na Atenção Básica com o Prontuário Eletrônico do Cidadão (PEC) do e-SUS AB, esclarecendo se as ações de integração permitiram aos trabalhadores e gestores locais descontinuarem o uso do respectivo SNIS. Foram entrevistadas três informantes-chave, sendo dois membros da equipe de desenvolvimento e gestão da Estratégia e-SUS AB no período de 2013 a 2017 e um membro da equipe técnica de uma entidade nacional representativa das secretarias municipais de saúde. A análise contou também com contribuições da experiência de um dos pesquisadores na utilização cotidiana do PEC/e-SUS AB e de outros SNIS a partir de seu trabalho como médico em uma unidade básica de saúde do Rio de Janeiro. Considerou-se como SNIS integrado apenas aqueles onde houve consenso entre as fontes acima.

Foi criada a seguinte tipologia para classificar a situação da integração entre sistemas:

- Interfaces de usuário integradas ou unificadas (integração completa): Quando dois ou mais sistemas se tornam invisíveis para o usuário final, ou seja, o usuário não consegue perceber que existe mais de um sistema em funcionamento, pois as interfaces estão totalmente integradas.
- Interfaces de usuário parcialmente integradas ou unificadas (integração incompleta): Quando foram identificadas iniciativas de integração entre as interfaces, mas ainda existe a necessidade, por motivos técnicos, políticos ou administrativos, dos profissionais e gestores continuarem utilizando os dois sistemas no cotidiano.
- Nenhuma integração ou unificação de interfaces de usuário: Quando não foi planejada nem realizada nenhuma ação para integração entre as interfaces dos sistemas.

Por fim, foi analisado o grau de integração dos SNIS com o organograma interno do MS. No período de 2013 a 2018, a estrutura formal do alto escalão do órgão se manteve estável, com a existência de sete Secretarias^26^: Atenção à Saúde (SAS), Executiva (SE), Vigilância em Saúde (SVS), Gestão Estratégica e Participativa (SGEP), Especial de Saúde Indígena (SESAI), Gestão do Trabalho e Educação na Saúde (SGTES) e Ciência Tecnologia e Insumos Estratégicos (SCTIE). O Departamento de Atenção Básica (DAB), principal setor responsável pela formulação e gestão da Estratégia e-SUS AB, encontrava-se vinculado à SAS.

A pesquisa foi apresentada e aprovada no Comitê de Ética do Hospital São Paulo da Universidade Federal de São Paulo (Unifesp), por meio do parecer nº 2.179.195. Os entrevistados assinaram Termo de Consentimento Livre e Esclarecido (TCLE) e as entrevistas foram realizadas e gravadas no software Skype^®^.

## RESULTADOS

Foram encontrados 31 SNIS em funcionamento na Atenção Básica entre 2013 e 2018, a maioria sob gestão técnica da SAS ou da SVS (Quadro).

**Quadro:** Sistemas Nacionais de Informação em Saúde (SNIS) que captaram dados na Atenção Básica entre 2013 a 2018 (excetuando-se os softwares da Estratégia e-SUS AB).

Sigla	Nome do sistema	Dado captado e/ou consumido ou principal finalidade na AB	Secretaria do MS
BFA	Sistema do Programa Bolsa Família na Saúde	Registro da Antropometria de beneficiários do Programa Bolsa família	SAS
Cadsus	Sistema de Cadastramento dos Usuários do SUS	Cadastro dos usuários do SUS	SE
CMD	Conjunto Mínimo de Dados	Registro de atividades e procedimentos ambulatoriais	SAS
CNES	Cadastro Nacional dos Estabelecimentos de Saúde	Cadastro dos estabelecimentos de saúde	SAS
Fique Sabendo	Sistema de controle dos Testes Rápidos de HIV, Sífilis e Hepatites Virais para ações estratégicas	Controle logístico dos testes rápidos	SVS
GAL	Sistema de Gestão de Ambiente Laboratorial	Visualização de Resultados de exames laboratoriais para doenças de interesse de saúde pública	SVS
Hiperdia	Sistema de Cadastramento e Acompanhamento de Hipertensos e Diabéticos	Monitoramento de pacientes com Hipertensão e Diabetes	SAS
Hórus	Sistema Nacional de Assistência Farmacêutica	Controle logístico de medicamentos do componente básico da Política Nacional de Assistência Farmacêutica	SVS
PMAQ-AB	Programa Nacional de Melhoria do Acesso e da Qualidade da Atenção Básica	Dados de ações programáticas e outras informações da Atenção Básica para fins de avaliação das equipes	SAS
Painel-PSE	Sistema de Adesão ao Programa Saúde na Escola	Monitoramento de atividades realizadas pelas equipes de Atenção Básica no âmbito do Programa Saúde na Escola	SAS
RAAS-AD	Registro das Ações de Saúde da Atenção Domiciliar	Registro de procedimentos da atenção domiciliar no âmbito do Programa Melhor em Casa	SAS
RESP	Resposta a Eventos de Saúde Pública	Notificação de casos microcefalia	SVS
SI-PNI	Sistema de informação do Programa Nacional de Imunização	Monitoramento de Cobertura vacinal e controle logístico de imunobiológicos	SVS
SIA	Sistema de informações ambulatoriais	Registro de procedimentos ambulatoriais	SAS
Siab	Sistema de Informação da Atenção Básica	Registro de atividades e procedimentos	SAS
Siasi	Sistema de Informação da Saúde Indígena	Demografia e acompanhamento clínico e administrativos da população indígena	SESAI
Siclom	Sistema de controle e monitoramento clínico e laboratorial dos pacientes em tratamento de hepatite	Prescrição de medicamentos antirretrovirais para tratamento de HIV/Aids	SVS
SIM	Sistema de Informações sobre Mortalidade	Registro de óbitos naturais	SVS
Sinan Net	Sistema de Informação de Agravos e Notificação	Notificação de doenças e agravos de interesse da saúde pública	SVS
Sinan Dengue/ Chikungunya	Sistema de Informação de Agravos de Notificação para Dengue e Chikungunya	Notificação de casos de dengue e febre Chikungunya	SVS
Sinan Influenza	Sistema de Informação de Agravos e Notificação – Influenza	Notificação de casos de Síndrome Respiratória Aguda Grave	SVS
Sinasc	Sistema de Informações sobre Nascidos Vivos	Registro de nascidos vivos	SVS
Siram	Sistema de Registro de Atendimento às Crianças com Microcefalia	Registro de dados assistenciais sobre crianças com diagnóstico de microcefalia	SAS
Siscan	Sistema de Informação do Câncer	Solicitação e resultados de pedidos de exames relacionados ao diagnóstico de câncer	SAS
Siscel	Sistema de Controle de Exames Laboratoriais de CD4 e Carga Viral	Pedidos de exames de CD4 e Carga Viral de pacientes com HIV	SVS
Sisreg	Sistema Nacional de Regulação	Solicitação e/ou marcação de procedimentos na atenção especializada	SAS
SIS Pré-natal	Sistema de Informações de Monitoramento e Avaliação do Pré-Natal, Parto, Puerpério e Criança	Acompanhamento de gestante, puérperas e recém-nascidos	SAS
Sisvan	Sistema de Vigilância e Acompanhamento Nutricional	Monitoramento do estado nutricional e consumo alimentar	SAS
Sivep DDA	Sistema Informatizado de Vigilância Epidemiológica de Doenças Diarreicas Agudas	Notificação de casos de Doença Diarreica Aguda	SVS
Sivep Malária	Sistema de Informação de Vigilância Epidemiológica de notificação de casos de malária	Notificação de casos de Malária	SVS
Telessaúde/Smart	Sistema de Monitoramento e Avaliação dos Resultados do Programa Telessaúde	Controle, monitoramento e avaliação nacional de programas de telessaúde	SGTES

MS: Ministério da Saúde; BFA: Sistema do Programa Bolsa Família na Saúde; Cadsus: Sistema de Cadastramento dos Usuários do SUS; CMD: Conjunto Mínimo de Dados; CNES: Cadastro Nacional dos Estabelecimentos de Saúde; GAL: Sistema de Gestão de Ambiente Laboratorial; Hiperdia: Sistema de Cadastramento e Acompanhamento de Hipertensos e Diabéticos; Hórus: Sistema Nacional de Assistência Farmacêutica; PMAQ-AB: Programa Nacional de Melhoria do Acesso e da Qualidade da Atenção Básica; Painel-PSE: Sistema de Adesão ao Programa Saúde na Escola; RAAS-AD: Registro de Ações de Saúde da Atenção Domiciliar; RESP: Resposta a Eventos de Saúde Pública; SI-PNI: Sistema de informação do Programa Nacional de Imunização; SIA: Sistema de informações ambulatoriais; Siab: Sistema de Informações da Atenção Básica; Siasi: Sistema de Informação da Saúde Indígena; Siclom: Sistema de Controle e Monitoramento Clínico e Laboratorial dos Pacientes em Tratamento de Hepatite; SIM: Sistema de Informação sobre Mortalidade; Sinan Net: Sistemas de Informação de Agravos de Notificação; Sinan Dengue/Chikungunya: Sistema de Informação de Agravos de Notificação para Dengue e Chikungunya; Sinasc: Sistema de Informações sobre Nascidos Vivos; Siram: Sistema de Registro de Atendimento às Crianças com Microcefalia; Siscan: Sistema de Informação do Câncer; Siscel: Sistema de Controle de Exames Laboratoriais de CD4 e Carga Viral; Sisreg: Sistema Nacional de Regulação; SIS Pré-natal: Sistema de Informações de Monitoramento e Avaliação do Pré-Natal, Parto, Puerpério e Criança. Sisvan: Sistema de Vigilância e Acompanhamento Nutricional; Sivep DDA: Sistema Informatizado de Vigilância Epidemiológica de Doenças Diarreicas Agudas; Sivep Malária: Sistema de Informação de Vigilância Epidemiológica de notificação de casos de malária; Telessaúde/Smart: Sistema de Monitoramento e Avaliação dos Resultados do Programa Telessaúde. SAS: Secretaria de Atenção à Saúde; SE: Secretaria Executiva; SVS: Secretaria de Vigilância em Saúde; SGEP: Secretaria de Gestão Estratégica e Participativa; SESAI: Secretaria Especial de Saúde Indígena; SGTES: Secretaria de Gestão do Trabalho e Educação na Saúde; SCTIE: Secretaria de Ciência Tecnologia e Insumos Estratégicos.

Verificou-se que a Estratégia e-SUS AB realizou unificação completa de interfaces de usuário com 12 dos 31 SNIS de base nacional em uso na Atenção Básica e integrações incompletas com quatro SNIS. Outros 15 SNIS não tiveram nenhum grau de integração, conforme apresentado na Tabela 1.

**Tabela 1:** Situação de integração de interfaces de usuário do e-SUS AB com outros SNIS.

Situação da integração	N SIS	%	Lista dos SIS
Completa	12	35,50%	Cadsus, CMD, Hiperdia, PMAQ-AB, Painel-PSE, RAAS-AD, SIA, Siab, Siram, SIS Pré-natal, Sisvan, BFA.
Incompleta	4	16,10%	CNES, Hórus, SI-PNI, Sisreg.
Nenhuma	15	48,40%	Fique Sabendo, GAL, RESP, Siasi, Siclom, SIM, Sinan Dengue/Chikungunya, Sinan Influenza, Sinan Net, Sinasc, Siscan, Siscel, Sivep Malária, Sivep DDA, Telessaúde/Smart.

MS: Ministério da Saúde; BFA: Sistema do Programa Bolsa Família na Saúde; Cadsus: Sistema de Cadastramento dos Usuários do SUS; CMD: Conjunto Mínimo de Dados; CNES: Cadastro Nacional dos Estabelecimentos de Saúde; GAL: Sistema de Gestão de Ambiente Laboratorial; Hiperdia: Sistema de Cadastramento e Acompanhamento de Hipertensos e Diabéticos; Hórus: Sistema Nacional de Assistência Farmacêutica; PMAQ-AB: Programa Nacional de Melhoria do Acesso e da Qualidade da Atenção Básica; Painel-PSE: Sistema de Adesão ao Programa Saúde na Escola; RAAS-AD: Registro de Ações de Saúde da Atenção Domiciliar; RESP: Resposta a Eventos de Saúde Pública; SI-PNI: Sistema de informação do Programa Nacional de Imunização; SIA: Sistema de informações ambulatoriais; Siab: Sistema de Informações da Atenção Básica; Siasi: Sistema de Informação da Saúde Indígena; Siclom: Sistema de Controle e Monitoramento Clínico e Laboratorial dos Pacientes em Tratamento de Hepatite; SIM: Sistema de Informação sobre Mortalidade; Sinan Net: Sistemas de Informação de Agravos de Notificação; Sinan Dengue/Chikungunya: Sistema de Informação de Agravos de Notificação para Dengue e Chikungunya; Sinasc: Sistema de Informações sobre Nascidos Vivos; Siram: Sistema de Registro de Atendimento às Crianças com Microcefalia; Siscan: Sistema de Informação do Câncer; Siscel: Sistema de Controle de Exames Laboratoriais de CD4 e Carga Viral; Sisreg: Sistema Nacional de Regulação; SIS Pré-natal: Sistema de Informações de Monitoramento e Avaliação do Pré-Natal, Parto, Puerpério e Criança. Sisvan: Sistema de Vigilância e Acompanhamento Nutricional; Sivep DDA: Sistema Informatizado de Vigilância Epidemiológica de Doenças Diarreicas Agudas; Sivep Malária: Sistema de Informação de Vigilância Epidemiológica de notificação de casos de malária; Telessaúde/Smart: Sistema de Monitoramento e Avaliação dos Resultados do Programa Telessaúde.

A integração variou conforme o setor interno do MS responsável pela gestão técnica do sistema (Tabela 2). Apesar da SAS e SVS terem números próximos de SNIS em uso na Atenção Básica, verificou-se uma importante disparidade entre a integração dos seus respectivos sistemas com o e-SUS AB.

**Tabela 2:** Integração do e-SUS AB com outros SNIS por Secretaria do MS. Organograma de setembro de 2018.

Secretaria do MS	Total de SNIS em usona Atenção Básica	Integração de SNIS com o e-SUS AB		
		Completa	Incompleta	Nenhuma
SAS	14	11	2	1
SVS	13	0	1	12
SE	1	1	0	0
SCTIE	1	0	1	0
SESAI	1	0	0	1
SGTES	1	0	0	1
SGEP	0	0	0	0
TOTAL	31	12	4	15

SAS: Secretaria de Atenção à Saúde; SE: Secretaria Executiva; SVS: Secretaria de Vigilância em Saúde; SGEP: Secretaria de Gestão Estratégica e Participativa; SESAI: Secretaria Especial de Saúde Indígena; SGTES: Secretaria de Gestão do Trabalho e Educação na Saúde; SCTIE: Secretaria de Ciência Tecnologia e Insumos Estratégicos.

## DISCUSSÃO

A fragmentação de SIS é um fenômeno relatado por diversos autores ao analisarem políticas de informação em saúde em diferentes regiões do mundo^27–31^, porém não foram identificados estudos que dimensionaram tal fragmentação a partir da contagem e descrição dos SNIS em funcionamento. O registro de 31 SNIS em uso na Atenção Básica no período de 2013 a 2018 evidencia que persiste no país a tendência de fragmentação, documentada no Brasil em diversos estudos técnicos^32–34^.

Nos países que apostaram em projetos de mudança desse cenário, o foco tem sido a melhor integração entre as bases de dados nacionais^35–37^ ou o estabelecimento de padrões semânticos e tecnológicos para registro e troca das informações entre diferentes sistemas^38,39^. Não foram encontrados estudos que descrevessem a integração de SNIS tendo como foco a integração de interfaces de usuário.

Quando analisamos o resultado da integração realizada pelo e-SUS AB e a estrutura político-organizacional do MS é possível notar que houve uma maior integração com os SNIS sob gestão direta do DAB (Figura) e uma baixa integração com sistemas das demais secretarias para além da própria SAS.

**Figura:** Integração de interfaces do e-SUS AB com SNIS utilizados na Atenção Básica conforme organograma interno do MS.

MS: Ministério da Saúde; SAS: Secretaria de Atenção à Saúde; DAB: Departamento de Atenção Básica; BFA: Sistema do Programa Bolsa Família na Saúde; Cadsus: Sistema de Cadastramento dos Usuários do SUS; CMD: Conjunto Mínimo de Dados; CNES: Cadastro Nacional dos Estabelecimentos de Saúde; GAL: Sistema de Gestão de Ambiente Laboratorial; Hiperdia: Sistema de Cadastramento e Acompanhamento de Hipertensos e Diabéticos; Hórus: Sistema Nacional de Assistência Farmacêutica; PMAQ-AB: Programa Nacional de Melhoria do Acesso e da Qualidade da Atenção Básica; Painel-PSE: Sistema de Adesão ao Programa Saúde na Escola; RAAS-AD: Registro de Ações de Saúde da Atenção Domiciliar; RESP: Resposta a Eventos de Saúde Pública; SI-PNI: Sistema de informação do Programa Nacional de Imunização; SIA: Sistema de informações ambulatoriais; Siab: Sistema de Informações da Atenção Básica; Siasi: Sistema de Informação da Saúde Indígena; Siclom: Sistema de Controle e Monitoramento Clínico e Laboratorial dos Pacientes em Tratamento de Hepatite; SIM: Sistema de Informação sobre Mortalidade; Sinan Net: Sistemas de Informação de Agravos de Notificação; Sinan Dengue/Chikungunya: Sistema de Informação de Agravos de Notificação para Dengue e Chikungunya; Sinasc: Sistema de Informações sobre Nascidos Vivos; Siram: Sistema de Registro de Atendimento às Crianças com Microcefalia; Siscan: Sistema de Informação do Câncer; Siscel: Sistema de Controle de Exames Laboratoriais de CD4 e Carga Viral; Sisreg: Sistema Nacional de Regulação; SIS Pré-natal: Sistema de Informações de Monitoramento e Avaliação do Pré-Natal, Parto, Puerpério e Criança. Sisvan: Sistema de Vigilância e Acompanhamento Nutricional; Sivep DDA: Sistema Informatizado de Vigilância Epidemiológica de Doenças Diarreicas Agudas; Sivep Malária: Sistema de Informação de Vigilância Epidemiológica de notificação de casos de malária; Telessaúde/Smart: Sistema de Monitoramento e Avaliação dos Resultados do Programa Telessaúde.

No caso da integração com os SNIS da SVS, que incluem as políticas de Vigilância Epidemiológica, de Atenção à População com HIV/Aids e Hepatites, de monitoramento da natalidade e mortalidade e de imunizações, houve avanço parcial apenas na integração do e-SUS AB com o SI-PNI.

O caso mais preocupante pareceu ser os SNIS do Departamento de HIV/Aids, que nem mesmo foram citados na lista de SNIS em funcionamento na Atenção Básica em licitação do MS para informatização de UBS^25^.

A disparidade entre a integração do e-SUS AB com os SNIS da SVS e da SAS pode ser considerada um sinal de que ainda persiste algum grau da histórica divisão e falsa dicotomia entre práticas e processos de Assistência à Saúde e Vigilância em Saúde^40^, mesmo após 30 anos da fundação do SUS e unificação das estruturas estatais da assistência hospitalar previdenciária e da saúde pública federal no MS.

A governabilidade da área gestora do e-SUS AB sobre outros SNIS se mostrou uma variável a ser considerada no processo de integração na medida em que houve uma maior taxa de sucesso de integração do e-SUS AB com os SNIS do próprio DAB quando comparada com os SNIS sob gestão de outros departamentos e secretarias do MS.

A frequência e intensidade do uso dos SNIS nos serviços de saúde demonstrou ser um fator de menor relevância, haja vista que os SNIS com uso intenso no nível local do SUS, como o Sisreg, Hórus e Sinan Net, tiveram integração incompleta ou não iniciada.

## CONCLUSÃO

Considerando a complexidade da integração entre sistemas legados nas organizações, é razoável que após cinco anos da implantação da Estratégia e-SUS AB, ele tenha se conectado apenas parcialmente com o total de SNIS em funcionamento na Atenção Básica. Apesar de ainda insuficiente, a integração de sistemas efetivada com cerca de ⅓ do SNIS em funcionamento da Atenção Básica, focada na redução de interfaces de usuário é um fato novo na agenda da política de informação e informática do SUS.

É notável a relativa singularidade da experiência brasileira de integração entre sistemas de informação em saúde. O Brasil apostou na criação de um prontuário eletrônico estatal como uma espécie de “hub” de outros SNIS criados pelo poder público, mais especificamente pelo Governo Federal. Apesar de consideramos que a postura “desenvolvimentista” assumida pelo Estado brasileiro contribui para a expansão da informatização da atenção básica no país, atentamos para a necessidade de maior integração do grande volume de software de mercado em utilização por estados e municípios nas bases nacionais dos SNIS, seguindo tendência de investimento em políticas de regulação pública associadas à maior interoperabilidade semântica e tecnológica entre os sistemas, conforme observado em outros países.

## Footnotes

a Controladoria-Geral da União (BR). Sistema Eletrônico do Serviço de Informação (e-SIC) e Fala.BR – Módulo de Acesso à Informação: acesso à informação e ouvidoria em uma única plataforma. Brasília, DF; 2021 [cited 2021 oct 20]. Available from: http://esic.cgu.gov.br
